# Topical Tacrolimus for Seborrhoeic Dermatitis: A Systematic Review of Efficacy and Indirect Malignancy Evidence

**DOI:** 10.7759/cureus.117866

**Published:** 2026-10-13

**Authors:** Ethel O Ojo, Success T Oyibo, Itawosim Orim, Sinmiloluwa Oke, Alberta Bow, Daniel E Onobun

**Affiliations:** 1 Internal Medicine, University Hospitals Birmingham NHS Foundation Trust, Birmingham, GBR; 2 Respiratory, Fairfield General Hospital, Greater Manchester, GBR; 3 Internal Medicine, Northampton General Hospital, Northampton, GBR; 4 Internal Medicine, University College London Hospitals NHS Foundation Trust, London, GBR; 5 Orthopaedics and Trauma, University Hospitals Coventry and Warwickshire NHS Trust, Coventry, GBR

**Keywords:** atopic eczema, cancer risk, seborrheic dermatitis, topical calcineurin inhibitors, topical tacrolimus

## Abstract

Seborrhoeic dermatitis (SD) is a chronic inflammatory skin disorder. Topical corticosteroids are effective for flares, but repeated facial use may thin the skin and cause telangiectasia or steroid-related facial eruptions. Topical tacrolimus is approved for atopic dermatitis (AD) in US and UK product labeling and is used off-label for SD. Its steroid-sparing potential must therefore be considered alongside the US Food and Drug Administration (FDA) boxed warning concerning a theoretical malignancy risk.

This systematic review evaluates the long-term efficacy, safety, and patient-reported outcomes associated with topical tacrolimus in SD, with particular focus on steroid-refractory and immunocompromised populations. Six databases were searched for English-language reports published from January 1, 2003, through June 5, 2025. Risk of bias was assessed using RoB 2, the Newcastle-Ottawa Scale (NOS), National Heart, Lung, and Blood Institute tools, or A MeaSurement Tool to Assess systematic Reviews (AMSTAR) 2 according to study design. Clinical and methodological heterogeneity precluded a defensible new meta-analysis, so results were synthesized narratively.

Twenty-one reports were included: 8 direct SD reports, 10 indirect primary safety, pharmacokinetic, or skin structure reports, and 3 prior systematic reviews. Maintenance evidence was more consistent than induction evidence. In the largest SD maintenance trial, relapse occurred in 12 of 57 tacrolimus recipients and 23 of 57 ciclopiroxolamine recipients (hazard ratio (HR): 0.44, 95% confidence interval (CI): 0.22-0.89). Transient burning or tingling was the principal adverse effect. No included SD study was large or long enough to assess malignancy. In AD, the largest synthesis reported a tacrolimus-specific odds ratio for any cancer of 0.99 (95% credible interval: 0.89-1.09), although some observational cohorts reported cutaneous T-cell lymphoma (CTCL) signals. Protopathic bias, disease severity, and surveillance are plausible explanations but do not exclude a causal association.

Topical tacrolimus appears useful for selected patients with facial SD, with the strongest direct evidence supporting twice-weekly maintenance after disease control. Long-term malignancy conclusions remain indirect for SD and should be communicated with appropriate uncertainty. Patient-preference and quality-of-life evidence is insufficient for firm conclusions.

## Introduction and background

Seborrhoeic dermatitis (SD) is a chronic, relapsing inflammatory dermatosis affecting sebaceous gland-rich areas, particularly the scalp, eyebrows, nasolabial folds, ears, and presternal skin. Its pathogenesis reflects interactions among *Malassezia* species, sebum, epidermal barrier function, and host inflammatory responses [1,2]. Facial visibility, scale, and pruritus can adversely affect quality of life and mental health [3].

Antifungal shampoos or creams and short courses of low-potency topical corticosteroids are commonly used, with treatment selected according to site and severity [1,4]. Repeated or prolonged corticosteroid use on the face may cause atrophy, telangiectasia, and steroid-related facial dermatoses [5]. These limitations create a need for nonatrophogenic anti-inflammatory options.

Tacrolimus binds FK506-binding protein and inhibits calcineurin-dependent T-cell activation and inflammatory cytokine transcription [6]. It is licensed for atopic dermatitis (AD), not SD, but small clinical studies and maintenance trials have evaluated it off-label in SD [7]. The US Food and Drug Administration (FDA) added a boxed warning to topical tacrolimus labeling in 2006 because of a theoretical malignancy risk derived from animal data, systemic immunosuppression, and postmarketing reports [8].

## Review

Methodology

Protocol and Registration

This review was prepared in accordance with Preferred Reporting Items for Systematic Reviews and Meta-Analyses (PRISMA) 2020 [9]. The protocol was not prospectively registered or publicly archived, and a dated, locked protocol and formal amendments log were not available. Retrospective registration was not undertaken because it would not constitute prospective registration. Ethical approval was not required because only published data were analyzed.

The PRISMA diagram is shown in Figure 1.

**Figure 1:**
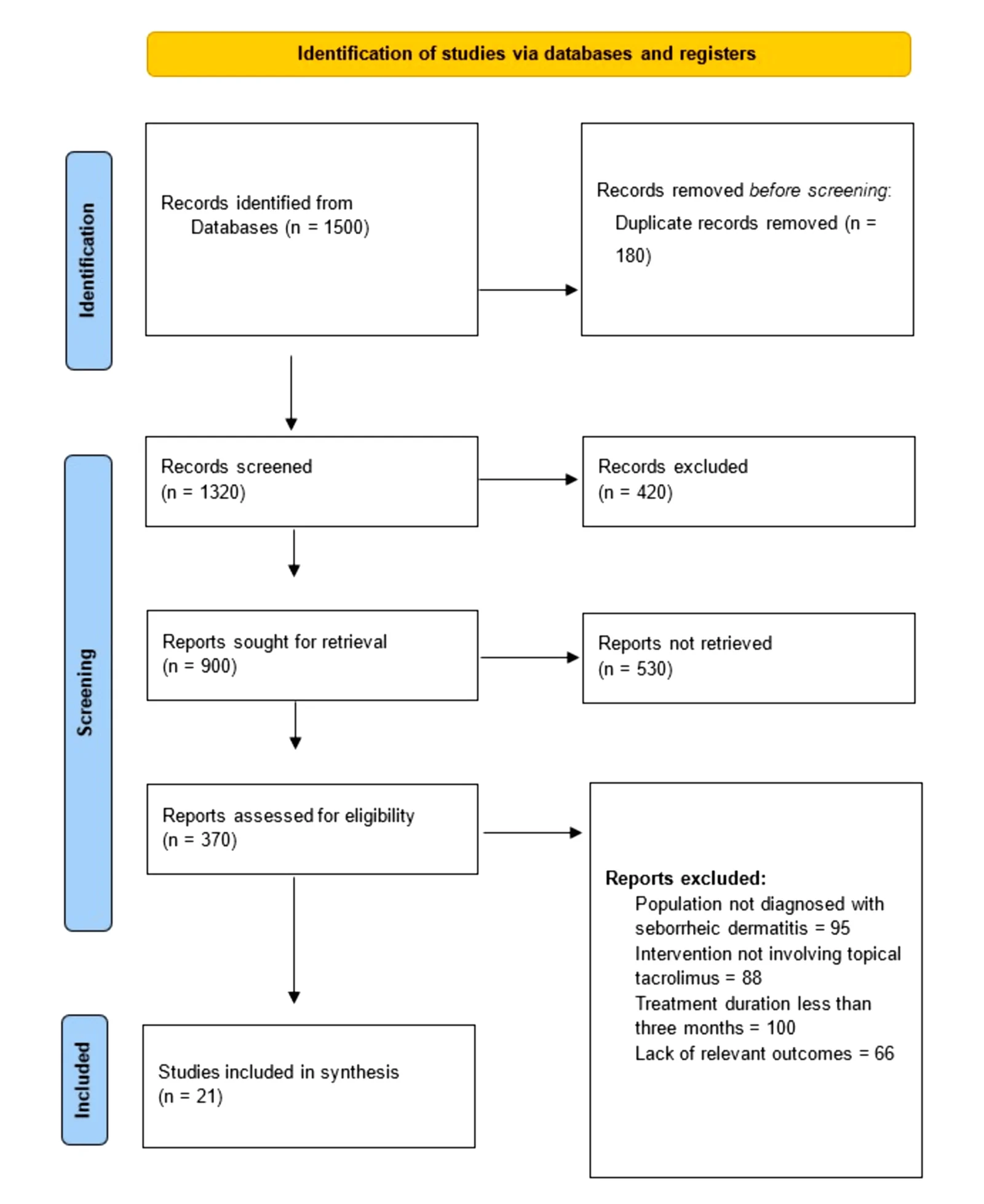
PRISMA flowchart PRISMA: Preferred Reporting Items for Systematic Reviews and Meta-Analyses

Eligibility Criteria

Two evidence streams were eligible. Direct SD evidence included randomized or nonrandomized clinical studies and case series with at least five participants who had clinically diagnosed SD and received topical tacrolimus 0.03% or 0.1%, regardless of treatment duration. Comparators could include vehicle, corticosteroids, antifungals, or another topical therapy. Indirect safety evidence included comparative cohorts, registries, pharmacokinetic studies, skin structure studies, and systematic reviews of topical tacrolimus or topical calcineurin inhibitors in dermatologic populations when malignancy, systemic exposure, or skin atrophy was reported. Animal and in vitro studies, oral or transplant tacrolimus, narrative reviews, isolated case reports, and reports without relevant outcomes were excluded from the main synthesis.

Information Sources and Search Strategy

PubMed/MEDLINE, Embase, Web of Science, Scopus, Cochrane CENTRAL, and ClinicalTrials.gov were searched for reports published from January 1, 2003, through June 5, 2025. Reference lists of relevant reviews were checked. The direct efficacy concept combined seborrhoeic dermatitis with tacrolimus, Protopic, or topical calcineurin inhibitor terms. The safety concept combined tacrolimus or topical calcineurin inhibitor terms with malignancy, lymphoma, melanoma, skin cancer, pharmacokinetic, absorption, or atrophy terms.

Only English-language full texts were included because translation resources were not available. This practical restriction may have excluded relevant studies and is treated as a potential source of language and publication bias.

Study Selection

Three reviewers screened titles and abstracts and assessed potentially eligible full texts; disagreements were resolved by consensus or consultation with a fourth reviewer. Duplicate records were managed in Mendeley. The direct and indirect streams were labeled during selection and remained separate throughout extraction, tabulation, synthesis, and interpretation.

Data Extraction

A standardized form captured country, design, population, sample size, tacrolimus concentration and schedule, comparator, follow-up, efficacy outcomes, adverse events, malignancy outcomes, and patient-reported outcomes. Extracted quantitative estimates were checked against the source reports before synthesis. The available audit record did not permit confirmation that every item underwent fully independent duplicate extraction; this is acknowledged as a limitation.

Risk-of-Bias Assessment

Randomized trials were evaluated using Cochrane RoB 2 [10]. Cohort and case-control studies were assessed using the Newcastle-Ottawa Scale (NOS) [11]. Uncontrolled intervention and pharmacokinetic studies were assessed using National Heart, Lung, and Blood Institute tools [12], and systematic reviews using A MeaSurement Tool to Assess systematic Reviews (AMSTAR) 2 [13]. Judgments were agreed by the review team, but a complete independent duplicate assessment record was not available. Directness was assessed separately: evidence from AD or eczema was rated indirect and was not used to establish efficacy or SD-specific cancer risk.

Data Synthesis and Certainty Assessment

Clinical and methodological heterogeneity in populations, comparators, schedules, outcomes, and designs precluded a defensible new pooled estimate. Studies were first grouped as direct SD evidence or indirect safety evidence. Direct studies were then organized as induction or maintenance treatment; indirect studies were organized as pharmacokinetic or skin structure evidence, primary malignancy cohorts, and prior systematic reviews. Effect estimates were reported as published and were not recalculated. Interpretation considered magnitude, precision, consistency, risk of bias, and directness rather than counting statistically significant results. No new I-squared statistic, funnel plot, Egger test, or subgroup meta-analysis was performed.

Results

Study Selection

The search identified 1,500 records. After removal of 180 duplicates, 1,320 titles and abstracts were screened and 420 were excluded. A total of 900 were sought for retrieval, with only full texts of 370 reports being assessed; 349 were excluded because the population, intervention, duration, or reported outcome did not meet eligibility criteria. Twenty-one reports entered the narrative synthesis (Figure 1).

Eight reports provided direct clinical evidence in SD, ten provided indirect primary safety, pharmacokinetic, or skin structure evidence, and three were prior systematic reviews (Figure 2). The included reports were published from 2003 through 2023 (Figure 3). The initial and extended Joint European Longitudinal Lymphoma and Skin Cancer Evaluation (JOELLE) publications represented the same cohort family and were not interpreted as independent replication.

**Figure 2:**
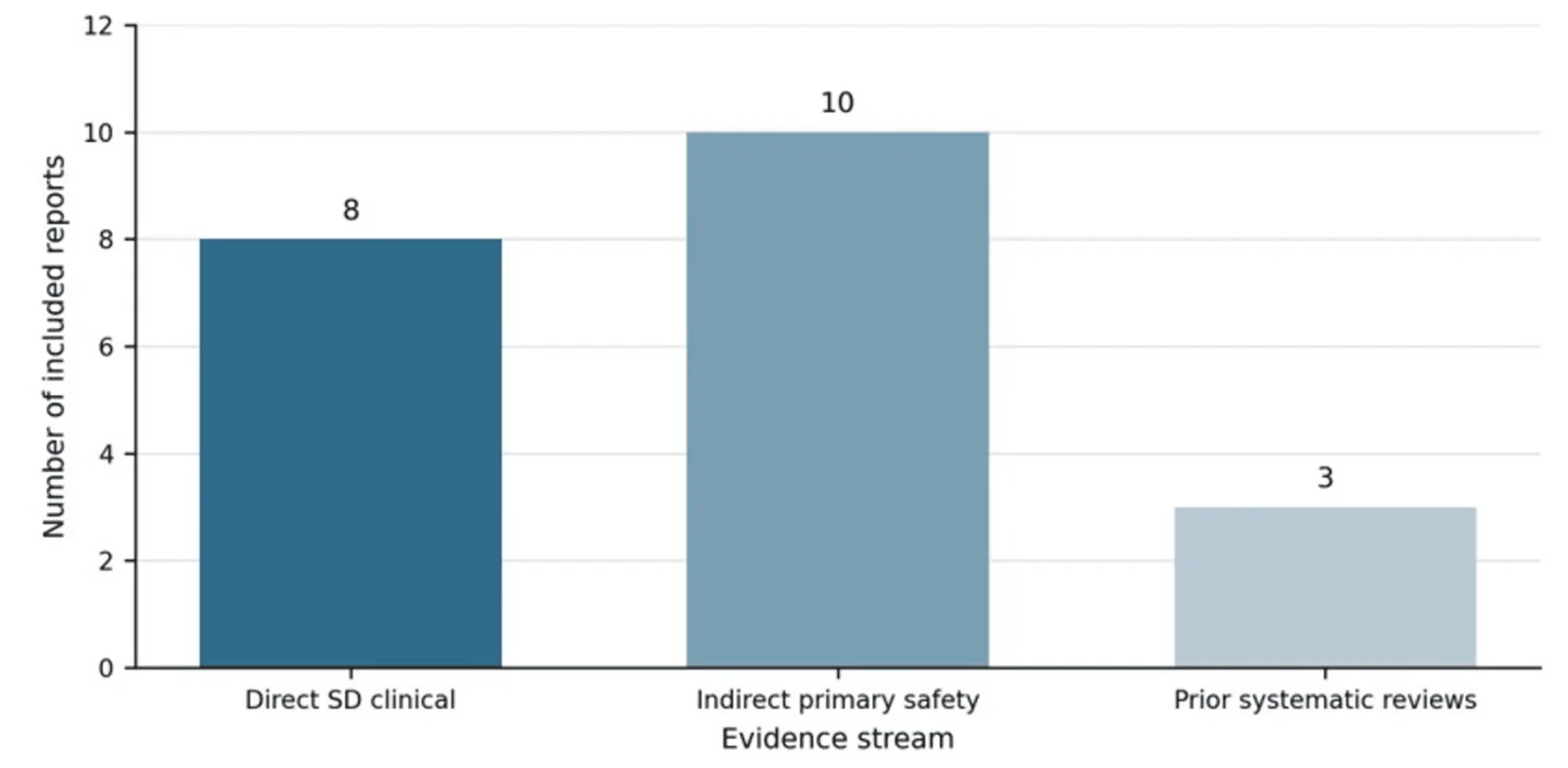
Included reports by evidence stream SD: seborrhoeic dermatitis

**Figure 3:**
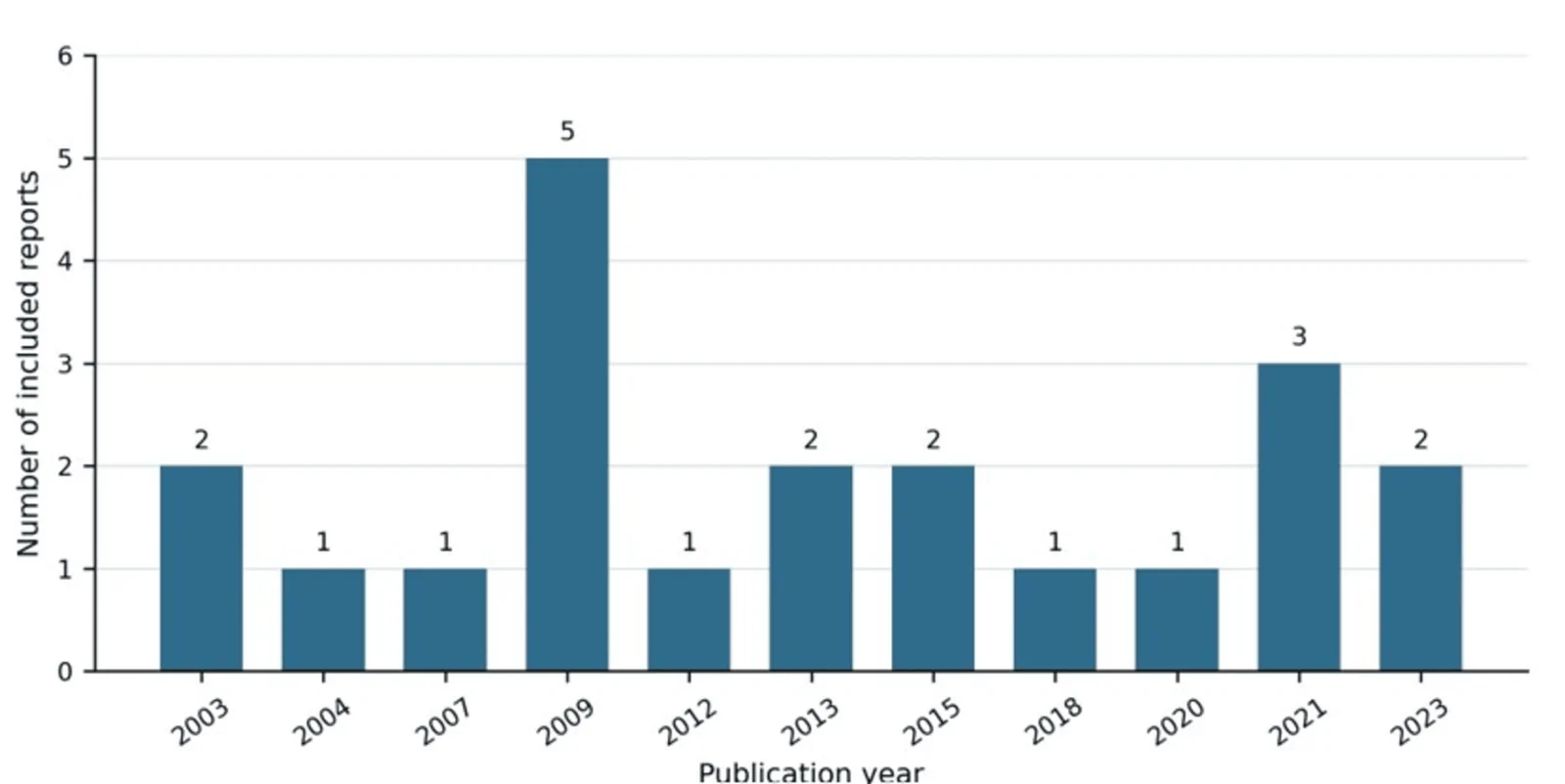
Publication year distribution of the included reports

Baseline Characteristics of Included Reports

The characteristics of the included studies are shown in Tables 1-3.

**Table 1:** Baseline characteristics of the eight direct seborrhoeic dermatitis clinical reports CI: confidence interval, SD: seborrhoeic dermatitis, SDASI-F: Seborrheic Dermatitis Area and Severity Index (Face)

Study	Setting/design/population	Intervention/comparator	Follow-up	Principal finding and limitation
Braza et al. (2003) [7]	USA; open-label pilot; adults with facial/non-scalp SD; n=16	Tacrolimus 0.1% nightly; no comparator	6 weeks	Erythema, scaling, and global scores improved; 13 completed; two reported transient pruritus/burning
Meshkinpour et al. (2003) [14]	USA; open-label pilot; consecutive patients with SD; n=18	Tacrolimus 0.1% twice daily; no comparator	Up to 28 days	Complete clearance in 61%; remaining participants had marked improvement; short, uncontrolled study
Shin et al. (2009) [15]	South Korea; randomized open comparative study; scalp SD; n=83	Tacrolimus 0.1% (n=27) versus betamethasone lotion (n=27) versus zinc pyrithione shampoo (n=29)	4-8 weeks	All groups improved at week 4; tacrolimus was similar to betamethasone; zinc pyrithione had more durable post-treatment improvement
Papp et al. (2012) [16]	Canada; single-blind randomized trial; adult facial SD; n=30	Tacrolimus 0.1% (n=16) versus hydrocortisone 1% (n=14), used for active disease	12 weeks	Similar SDASI-F improvement (P=0.86); tacrolimus required fewer applications; three treatment-related local events in tacrolimus arm
Goldust et al. (2013) [17]	Iran; comparative clinical trial; SD; n=60	Tacrolimus 0.03% (n=30) versus sertaconazole 2% (n=30), twice daily	4 weeks	Both improved; day-28 satisfaction was 83.3% with tacrolimus and 90% with sertaconazole; reporting of randomization/blinding was limited
Kim et al. (2013) [18]	South Korea; multicenter double-blind randomized maintenance trial; facial SD; 104 induction, 75 randomized	Tacrolimus 0.1% once weekly (n=28), twice weekly (n=32), or vehicle twice weekly (n=15)	2-week induction + 10-week maintenance	Both tacrolimus schedules outperformed vehicle; twice-weekly treatment produced the lowest recurrence; six withdrew across phases for burning/tingling
Kim et al. (2015) [19]	South Korea; prospective open-label multicenter maintenance study; facial SD; 123 induction, 83 maintenance	Tacrolimus 0.1% twice daily to stabilize, then twice weekly; AD comparison and historical vehicle data	Up to 4-week induction + 20-week maintenance	Fifty-four of 83 patients with SD (65%) had no exacerbation during maintenance; no concurrent randomized SD control
Joly et al. (2021) [20]	France; multicenter double-blind randomized maintenance trial; severe facial SD; n=114	Tacrolimus 0.1% versus ciclopirox olamine 1%, each twice weekly after desonide induction (57 per arm)	24 weeks	Relapse in 12 versus 23; hazard ratio: 0.44 (95% CI: 0.22-0.89); planned sample size was not reached

**Table 2:** Characteristics of the 10 indirect primary safety, pharmacokinetic, or skin structure reports AD: atopic dermatitis, APPLES: A Prospective Pediatric Longitudinal Evaluation to Assess the Long-Term Safety of Tacrolimus Ointment for the Treatment of Atopic Dermatitis, CI: confidence interval, CTCL: cutaneous T-cell lymphoma, HR: hazard ratio, IRR: incidence rate ratio, OD: odds ratio, SD: seborrhoeic dermatitis, SIR: standardized incidence ratio, TCI: topical calcineurin inhibitor, THIN: The Health Improvement Network

Study	Setting/design	Population	Follow-up	Principal finding and directness
Undre et al. (2009) [21]	Ireland; pharmacokinetic AD study	Adults, n=14; 11 analyzed	2 weeks	Systemic exposure was low; 94% of blood samples were <1 ng/mL; small and indirect for SD
Kyllönen et al. (2004) [22]	Finland; prospective open comparative AD study	Tacrolimus, n=56; steroid-treated AD, n=36; healthy, n=27	12 months	Tacrolimus increased collagen markers and skin thickness; directness to SD is limited
Arellano et al. (2007) [23]	USA claims; nested case-control in AD	Cohort, n=293,253; lymphoma cases, n=294	Retrospective	Tacrolimus OR: 0.8 (95% CI: 0.4-1.7); AD severity was the principal lymphoma correlate
Hui et al. (2009) [24]	USA health system; retrospective cohort in AD/eczema	n=953,064	2001-2004 exposure window	No overall cancer increase; CTCL signal (HR: 5.04); chart review suggested possible pre-existing CTCL in some exposed cases
Schneeweiss et al. (2009) [25]	USA claims; propensity-matched cohort	92,585 pimecrolimus initiators plus tacrolimus, corticosteroid, and untreated cohorts	Mean: 1.3 years	No excess lymphoma versus active topical comparators; short latency limits cancer inference
Arellano et al. (2009) [26]	UK THIN; nested case-control	Source database about 3.5 million; TCI exposure uncommon	Retrospective	No lymphoma cases occurred among TCI users; study chiefly associated lymphoma with AD severity and potent corticosteroid exposure
Castellsague et al. (2018) [27]	Denmark, Sweden, UK, Netherlands; matched JOELLE cohort	Tacrolimus initiators: 19,948 children and 66,127 adults	Variable; initial JOELLE analysis	Some lymphoma/CTCL signals; absolute incidence low and residual confounding, surveillance and reverse causation plausible
Paller et al. (2020) [28]	International prospective pediatric registry (APPLES)	Children with AD exposed to tacrolimus; n=7,954	Up to 10 years	Six cancers; SIR: 1.01 (95% CI: 0.37-2.20); no lymphomas
Arana et al. (2021) [29]	Four-country JOELLE extension; matched cohort	Adult tacrolimus, n=126,908; children, n=32,605	At least 10 years for 19% of adults and 32% of children	Adult CTCL IRR: 1.80 (95% CI: 1.25-2.58); other lymphoma/skin cancer estimates mostly null; long-latency estimates did not increase
Huang et al. (2023) [30]	Taiwan national claims; propensity-matched AD cohort	TCI, n=39,185; corticosteroid comparator, n=156,740	Retrospective	No association with overall cancer, lymphoma, or skin cancer; leukemia signal warrants cautious interpretation

**Table 3:** Characteristics of the three prior systematic reviews used as secondary safety evidence AD: atopic dermatitis, OR: odds ratio

Study	Design	Evidence base	Principal finding	Limitation
Legendre et al. (2015) [31]	Systematic review/meta-analysis	22 studies of AD, lymphoma, and topical treatment	AD severity was associated with lymphoma; topical treatment was not clearly causal	Indirect to SD; earlier evidence base
Lam et al. (2021) [32]	Systematic review/meta-analysis	11 observational studies; 408,366 treated participants plus comparators	No overall or skin cancer association; lymphoma RR elevated but absolute risk low	Susceptible to confounding and protopathic bias in included studies
Devasenapathy et al. (2023) [33]	Systematic review/Bayesian meta-analysis	110 unique AD studies; 3.4 million patients	Any cancer OR 1.03 (95% credible interval: 0.94-1.11); tacrolimus OR: 0.99 (0.89-1.09)	Moderate-certainty evidence; mean follow-up: 11 months

Direct SD Efficacy and Tolerability

The two 2003 uncontrolled pilots reported short-term improvement with tacrolimus 0.1%, but both were small and were rated fair using the National Institutes of Health (NIH) tool [7,14]. In the open scalp study, rated as having some concerns with RoB 2, tacrolimus and betamethasone groups both improved at week 4 and worsened after discontinuation, whereas zinc pyrithione continued to improve [15]. These observations support anti-inflammatory activity but do not establish superiority, equivalence, or non-inferiority.

In the 12-week facial SD trial, which had some RoB 2 concerns, the observed Seborrheic Dermatitis Area and Severity Index (Face) (SDASI-F) changes were similar with tacrolimus and hydrocortisone (P=0.86), and tacrolimus was used on fewer days [16]. The study was not designed or powered as an equivalence or non-inferiority trial. A four-week comparison at high risk of bias reported improvement with tacrolimus 0.03% and sertaconazole; satisfaction numerically favored sertaconazole (90% versus 83.3%) [17].

Maintenance evidence was more consistent. The Korean trial, rated high risk because of responder enrichment and attrition, found once- and twice-weekly tacrolimus better maintained control than vehicle, with the lowest recurrence in the twice-weekly group [18]. In a fair-quality open prospective study, 54 of 83 participants with SD (65%) had no exacerbation during 20 weeks of twice-weekly maintenance [19]. The low-risk French trial found relapse in 12 of 57 tacrolimus recipients and 23 of 57 ciclopiroxolamine recipients (hazard ratio: 0.44, 95% confidence interval: 0.22-0.89) [20].

Application site burning, tingling, pruritus, flushing, or irritation were the main adverse effects. Events were usually transient, although six participants withdrew across induction and maintenance in the Korean randomized study because of burning or tingling [18]. No direct SD trial was sufficiently large or long to evaluate malignancy.

Indirect Safety and Malignancy Evidence

The following evidence is indirect for SD. In a small, fair-quality pharmacokinetic study of adults with AD, 94% of blood tacrolimus concentrations were below 1 ng/mL [21]. A fair-quality, one-year nonrandomized AD study found increased collagen synthesis and skin thickness with tacrolimus [22]. These findings support biological plausibility for a steroid-sparing approach but do not establish long-term SD safety.

Cancer findings were heterogeneous. Arellano et al. reported an adjusted lymphoma odds ratio of 0.8 (95% confidence interval: 0.4-1.7) and found AD severity to be a stronger correlate [23]. Hui et al. found no overall cancer increase but reported a T-cell lymphoma association; chart review suggested that some lesions predated exposure [24]. Schneeweiss et al. reported similar lymphoma rates across topical treatment initiators, but the mean follow-up was only 1.3 years [25]. The UK The Health Improvement Network (THIN) study had too few exposed cases for a stable tacrolimus estimate [26]. NOS scores were 6/9 to 7/9 for these studies, with residual confounding and outcome classification limitations.

The initial JOELLE study reported low absolute rates but some lymphoma and cutaneous T-cell lymphoma (CTCL) signals [27]. A Prospective Pediatric Longitudinal Evaluation to Assess the Long-Term Safety of Tacrolimus Ointment for the Treatment of Atopic Dermatitis (APPLES) observed six cancers and no lymphomas among 7,954 children followed for up to 10 years (standardized incidence ratio: 1.01, 95% confidence interval: 0.37-2.20) [28]. In the higher-quality JOELLE extension (NOS 8/9), adult tacrolimus use was associated with CTCL (incidence rate ratio: 1.80, 95% confidence interval: 1.25-2.58), whereas estimates for melanoma, non-Hodgkin lymphoma, and Hodgkin lymphoma were below 1 and estimates after at least five years did not increase [29]. A Taiwanese cohort (NOS 7/9) found no association with overall cancer, lymphoma, or skin cancer; a leukemia signal requires replication [30].

Prior syntheses were broadly reassuring for overall cancer but not identical for lymphoma. Legendre et al. emphasized the association between AD severity and lymphoma [31]. Lam et al. found no association with cancer overall or skin cancer but a small relative increase in lymphoma, with low absolute risk [32]. Devasenapathy et al., the highest-confidence review, analyzed 110 unique studies and 3.4 million patients with AD: the odds ratio for any cancer was 1.03 (95% credible interval: 0.94-1.11), and the tacrolimus-specific odds ratio was 0.99 (0.89-1.09) [33]. These non-significant estimates do not prove absence of risk, particularly for rare outcomes or for SD.

Patient-Reported Outcomes

Patient-reported evidence was sparse. Papp et al. included patient global assessment and observed less frequent application with tacrolimus [16]. Goldust et al. reported satisfaction but did not favor tacrolimus over sertaconazole [17]. No included direct SD study reported a Dermatology Life Quality Index (DLQI) analysis. The evidence does not support a general claim that patients prefer tacrolimus.

Risk of Bias and Certainty of Evidence

Study-level judgments are presented in Table 4. Only the Joly trial was judged low risk overall. The remaining direct SD evidence had some concerns, high risk, or fair quality because of small samples, open treatment, incomplete allocation reporting, attrition, and responder enrichment. Observational cancer studies were large but could not fully separate treatment from disease severity, surveillance, or early misdiagnosed CTCL. Outcome-level certainty was moderate for maintenance benefit, low for short-term comparative efficacy and local tolerability, very low for patient-reported benefit, moderate for overall cancer association within AD, and very low for SD-specific malignancy inference (Table 5).

**Table 4:** Study-level risk-of-bias and methodological-quality judgments RoB 2 uses low, some concerns, and high; NOS is reported out of nine stars; NIH and AMSTAR 2 judgments are qualitative. AMSTAR 2: A MeaSurement Tool to Assess systematic Reviews 2, NIH: National Institutes of Health, NOS: Newcastle-Ottawa Scale, TCI: topical calcineurin inhibitor

Study	Tool	Key concern or strength	Overall appraisal
Braza et al. (2003) [7]	NIH uncontrolled intervention	No comparator; small sample; 3/16 lost by week 6	Fair
Meshkinpour et al. (2003) [14]	NIH uncontrolled intervention	No comparator; short follow-up; consecutive recruitment	Fair
Shin et al. (2009) [15]	RoB 2	Open label; allocation concealment unclear; attrition at week 8	Some concerns
Papp et al. (2012) [16]	RoB 2	Small single-center trial; participants unblinded, assessor blinded	Some concerns
Goldust et al. (2013) [17]	RoB 2	Randomization, concealment, and blinded assessment insufficiently reported	High
Kim et al. (2013) [18]	RoB 2	Responder-enriched maintenance population; 19/75 randomized participants did not complete	High
Kim et al. (2015) [19]	NIH uncontrolled intervention	Open label; historical vehicle comparison; attrition	Fair
Joly et al. (2021) [20]	RoB 2	Double-blind multicenter trial; target sample size not reached	Low
Undre et al. (2009) [21]	NIH uncontrolled intervention	Very small sample; 11/14 analyzed; objective assays	Fair
Kyllönen et al. (2004) [22]	NIH uncontrolled/comparative	Open allocation and nonrandomized control groups; objective biomarkers	Fair
Arellano et al. (2007) [23]	NOS	Claims exposure/outcome; severity adjusted but residual confounding remains	7/9
Hui et al. (2009) [24]	NOS	Outcome validation differed by exposure; strong risk of protopathic bias	6/9
Schneeweiss et al. (2009) [25]	NOS	Active comparators and propensity methods; mean follow-up only 1.3 years	7/9
Arellano et al. (2009) [26]	NOS	Validated outcomes; TCI exposure too sparse for a precise TCI estimate	7/9
Castellsague et al. (2018) [27]	NOS	Large active comparator cohort; residual severity and reverse causation concerns	7/9
Paller et al. (2020) [28]	NOS	Prospective registry; external comparator and attrition limit causal inference	6/9
Arana et al. (2021) [29]	NOS	Large, matched, extended follow-up; few pediatric events and residual confounding	8/9
Huang et al. (2023) [30]	NOS	Large propensity-matched claims cohort; exposure misclassification and residual confounding	7/9
Legendre et al. (2015) [31]	AMSTAR 2	Earlier search and incomplete control of overlap/confounding	Moderate confidence
Lam et al. (2021) [32]	AMSTAR 2	Transparent duplicate methods; observational evidence remains confounded	Moderate confidence
Devasenapathy et al. (2023) [33]	AMSTAR 2	Protocol, broad search, duplicate appraisal and certainty assessment reported	High confidence

**Table 5:** Outcome-level certainty assessment AD: atopic dermatitis, CTCL: cutaneous T-cell lymphoma, DLQI: Dermatology Life Quality Index, SD: seborrhoeic dermatitis

Outcome	Evidence base	Certainty	Main reason for rating
Short-term improvement in SD	Eight direct reports; mainly small or uncontrolled; active comparators varied	Low	Risk of bias, imprecision, and lack of trials designed for equivalence or non-inferiority
Prevention of relapse after disease control	Two randomized maintenance trials plus one prospective maintenance study	Moderate	Consistent maintenance benefit, including HR of 0.44, but modest samples and responder enrichment
Local tolerability	Direct SD studies	Low	Burning or tingling was consistently reported, but event definitions and ascertainment varied
Overall cancer association with topical tacrolimus	Large AD cohorts and prior systematic reviews	Moderate for AD; very low for SD-specific inference	Large evidence base but observational confounding and serious indirectness for SD
CTCL or lymphoma causality	Observational cohorts and prior reviews	Low	Rare outcomes, inconsistent signals, surveillance, severity confounding, and possible protopathic bias
Patient satisfaction or quality of life	Two direct studies with limited patient-reported measures	Very low	Sparse reporting; no included direct study reported DLQI

Discussion

Principal Findings

This systematic review suggests that topical tacrolimus has a clinically useful but relatively specific role in the management of seborrhoeic dermatitis (SD). The available evidence is most convincing for maintenance treatment in patients with recurrent facial disease after initial disease control, rather than for demonstrating superiority during treatment of an acute flare. Across the direct SD literature, tacrolimus was associated with improvements in erythema, scaling, and pruritus; however, heterogeneity in treatment duration, comparator therapy, outcome definitions, and study design limits the strength of comparative conclusions [14-20]. Several studies were also small, uncontrolled, or insufficiently powered to demonstrate equivalence or non-inferiority.

These findings are broadly consistent with the wider literature evaluating topical anti-inflammatory therapies for SD. A Cochrane review of topical anti-inflammatory agents found that topical corticosteroids and calcineurin inhibitors can improve symptoms of facial and scalp SD, but emphasized that most trials were small and of short duration and that evidence regarding longer-term disease control was limited [34]. Similarly, a systematic review focused specifically on facial SD identified calcineurin inhibitors, antifungal agents, and corticosteroids as effective topical options, while highlighting substantial heterogeneity between trials and the recurrent nature of the disease [35].

This distinction between short-term improvement and maintenance of remission is clinically important. SD is a chronic, relapsing disorder rather than an illness in which short-term lesion clearance necessarily represents durable therapeutic success [1,2]. Consequently, an intervention that reduces the frequency of relapse or delays the need for further corticosteroid treatment may provide clinically meaningful benefit even where its acute efficacy is not superior to existing treatments. The present findings therefore support consideration of topical tacrolimus as part of a longer-term steroid-sparing strategy in appropriately selected patients rather than as a universal replacement for established antifungal or corticosteroid treatment.

Efficacy and Maintenance Treatment

The strongest direct evidence relates to proactive maintenance therapy. Kim et al. demonstrated that intermittent tacrolimus 0.1% maintained improvements in erythema, scaling, and pruritus following initial disease stabilization, with twice-weekly treatment producing better disease control than once-weekly treatment or vehicle [18]. An additional prospective multicenter study reported prolonged control of facial SD using twice-weekly tacrolimus maintenance for up to 20 weeks [19]. These observations suggest that continued intermittent suppression of inflammation after visible improvement may help reduce recurrence rather than simply treating successive flares.

The multicenter, double-blind randomized trial by Joly et al. provides the most clinically informative evidence supporting this approach [20]. Following initial control with desonide, patients with severe facial SD were randomized to twice-weekly tacrolimus 0.1% or ciclopiroxolamine 1%. Relapse occurred in 12 of 57 patients receiving tacrolimus compared with 23 of 57 receiving ciclopiroxolamine, corresponding to a hazard ratio of 0.44 (95% CI: 0.22-0.89). Because the comparator was an active topical treatment rather than vehicle, this study strengthens the case for tacrolimus as a maintenance option after successful induction.

The concept of proactive intermittent anti-inflammatory therapy is also supported indirectly by experience in atopic dermatitis (AD). In a 12-month randomized study, twice-weekly tacrolimus 0.1% applied to previously affected areas reduced exacerbations and prolonged the time to first flare compared with vehicle [36]. Although AD and SD have distinct pathophysiological features and this evidence should not be interpreted as direct evidence for SD efficacy, it provides biological and therapeutic context for the proactive treatment strategy evaluated in the SD maintenance studies.

The apparent benefit of maintenance treatment is plausible within the current understanding of SD pathogenesis. SD reflects a complex interaction between *Malassezia* species, sebaceous activity, epidermal barrier function, and host inflammatory responses [1,2]. Resolution of visible erythema and scaling may therefore not represent complete resolution of the inflammatory susceptibility underlying recurrent disease. Intermittent anti-inflammatory treatment may suppress residual or recurrent inflammation before it becomes clinically apparent. However, mechanistic inference should remain cautious because the included clinical studies were not designed to demonstrate persistence of subclinical inflammation or establish the mechanism through which maintenance tacrolimus reduces relapse.

Importantly, the evidence does not demonstrate that tacrolimus is universally superior to conventional acute treatment. In the trial by Papp et al., both tacrolimus 0.1% and hydrocortisone 1% produced improvement in facial SD, with no significant difference in the primary severity outcome, although tacrolimus was used on fewer treatment days [16]. The study was not designed to establish formal therapeutic equivalence. Other comparisons with corticosteroids, zinc pyrithione, and sertaconazole also produced heterogeneous results [15,17]. This is consistent with the Cochrane review, which concluded that calcineurin inhibitors appear effective but that the available comparative evidence is limited by short follow-up, small sample sizes, and heterogeneity [34]. Accordingly, the clinical value of tacrolimus may lie less in superior acute efficacy than in its potential utility for recurrent disease in anatomically sensitive areas.

Steroid-Sparing Role and Tolerability

The steroid-sparing potential of tacrolimus is particularly relevant in facial SD. Topical corticosteroids are effective anti-inflammatory treatments and remain appropriate for short courses during symptomatic flares. Repeated or prolonged exposure on facial skin, however, can contribute to cutaneous atrophy, telangiectasia, and steroid-associated dermatoses [5]. Tacrolimus acts through calcineurin inhibition rather than glucocorticoid receptor signaling [6] and does not share the same mechanism of corticosteroid-induced skin atrophy. The skin structure evidence identified in this review provides indirect support for preservation of dermal collagen synthesis and skin thickness during prolonged tacrolimus exposure [22].

This may be clinically important for recurrent involvement of the eyelids, nasolabial folds, and other facial areas where repeated courses of corticosteroids may be undesirable. The wider review literature on facial SD similarly recognizes topical calcineurin inhibitors as useful alternatives in recurrent inflammatory disease, particularly where avoidance of prolonged corticosteroid exposure is desirable [34,35]. Evidence from pimecrolimus trials provides additional class-level support for the effectiveness of topical calcineurin inhibition in facial SD, although pimecrolimus and tacrolimus should not be assumed to have identical efficacy or safety profiles [37].

This potential advantage must be balanced against local tolerability. Burning, tingling, warmth, pruritus, and irritation were the most frequently reported adverse effects in the included tacrolimus studies [14-20]. These reactions were generally transient, although treatment discontinuation occurred in a minority of participants. Local application site symptoms are also well recognized across the wider topical calcineurin inhibitor literature [33,34]. Accordingly, patients should be counseled before initiation that transient burning or warmth may occur, particularly when treatment is first applied to actively inflamed skin.

This counseling may have practical importance because anticipated and self-limited application site discomfort may otherwise be interpreted as allergy, worsening disease, or treatment failure. Nevertheless, the direct SD literature provides relatively little information on how local adverse effects influence adherence or longer-term treatment persistence. This represents an important evidence gap, particularly for a treatment proposed primarily as maintenance therapy.

Long-Term Safety and Malignancy Evidence

The question of malignancy requires a different evidential framework from short-term treatment efficacy. The US Food and Drug Administration introduced a boxed warning for topical tacrolimus and pimecrolimus in 2006, based on a theoretical malignancy risk arising from animal data, experience with systemic immunosuppression, and postmarketing reports [8]. The warning remains important in clinical counseling and may influence both clinician and patient willingness to use topical tacrolimus for prolonged periods.

However, topical exposure differs substantially from systemic calcineurin inhibition. Pharmacokinetic evidence included in this review demonstrated low systemic tacrolimus concentrations following conventional topical treatment, with 94% of blood samples in one AD study having tacrolimus concentrations below 1 ng/mL [21]. These findings support the biological distinction between topical and systemic exposure, although low systemic absorption alone cannot establish long-term oncological safety.

No eligible SD study was sufficiently large or prolonged to measure malignancy incidence. Therefore, the absence of malignancies in the direct SD studies should not be interpreted as evidence that long-term malignancy risk is absent. Instead, assessment of this outcome necessarily relies on indirect evidence, predominantly from considerably larger AD populations.

The largest contemporary synthesis is broadly reassuring regarding overall cancer risk. Devasenapathy et al. included 110 unique studies and approximately 3.4 million patients and found little to no association between topical calcineurin inhibitor exposure and cancer overall; the tacrolimus-specific odds ratio was 0.99 (95% credible interval: 0.89-1.09) [33]. These data provide considerably greater reassurance regarding overall malignancy than was available when the boxed warning was introduced. Nevertheless, they derive predominantly from AD populations and should not be represented as direct evidence of long-term safety in SD.

The interpretation of lymphoma and particularly cutaneous T-cell lymphoma (CTCL) is more complex. Positive associations have been reported in some observational datasets [24,27,29,32]. In the extended JOELLE analysis, for example, adult topical tacrolimus users demonstrated an increased estimate for CTCL while estimates for several other malignancies were closer to the null [29]. These signals require acknowledgement, but their causal interpretation is difficult.

Several mechanisms of bias are particularly relevant. First, early CTCL can resemble eczema or other inflammatory dermatoses. Tacrolimus may therefore be prescribed to lesions that are subsequently recognized as CTCL, creating protopathic bias in which the early manifestations of malignancy precede rather than follow exposure. Second, patients with more severe or persistent inflammatory skin disease are more likely to receive second-line therapies such as tacrolimus, introducing confounding by indication. Disease severity itself has been associated with lymphoma risk in previous analyses [31]. Third, patients receiving prescription immunomodulatory therapy may undergo more frequent dermatologic assessment, increasing opportunities for detection compared with less intensively monitored patients.

The longer-term JOELLE data are therefore informative. The absence of progressively increasing risk estimates with increasing exposure latency weakens, although does not exclude, a straightforward cumulative carcinogenic explanation [29]. Similarly, previous systematic reviews have found no clear increase in overall cancer or skin cancer while reporting greater uncertainty around lymphoma [31-33]. Taken together, these data are reassuring regarding overall malignancy but do not justify stating that all cancer risk has been definitively excluded. In particular, rare outcomes such as CTCL remain challenging to evaluate because even very large observational datasets may be affected by diagnostic misclassification, confounding, and protopathic bias.

Applicability of Indirect Evidence to Seborrhoeic Dermatitis

Extrapolation from AD to SD deserves explicit consideration. Patients with AD may differ from those with SD in age distribution, extent of barrier dysfunction, total treated body surface area, intensity and duration of topical treatment, background inflammatory activity, and use of other immunomodulatory therapies. Consequently, AD safety datasets are useful for assessing the consequences of topical calcineurin inhibitor exposure in general but cannot establish the magnitude of long-term malignancy risk specifically in SD.

The distinction is especially important because typical facial SD may involve a relatively small body surface area and intermittent exposure, potentially resulting in a different cumulative exposure profile from moderate-to-severe AD. Conversely, the generally milder nature of SD means that even a very small treatment-related risk could influence the risk-benefit balance differently. The certainty of the SD-specific malignancy inference therefore remains very low despite much stronger evidence regarding overall cancer risk in the broader AD population.

Clinical Implications

The results support a focused rather than universal role for topical tacrolimus. Antifungal therapy and short courses of low-potency corticosteroids remain established treatments for SD [1,4,34,35]. Tacrolimus should therefore not be considered a routine replacement for these agents in all patients. Its clearest potential role is in recurrent facial SD requiring repeated anti-inflammatory treatment, particularly where cumulative corticosteroid exposure is undesirable or where conventional therapy has provided inadequate long-term disease control.

A pragmatic treatment pathway supported by the available evidence is initial control of an active flare followed, in selected patients with frequent recurrence, by proactive intermittent maintenance. Among the regimens evaluated directly in SD, tacrolimus 0.1% twice weekly has the strongest evidence [18-20]. Treatment decisions should nevertheless incorporate disease severity, anatomical site, previous treatment response, local tolerability, patient preference, and the off-label status of tacrolimus for SD.

Patients should also be informed of the distinction between the FDA boxed warning and the contemporary evidence base. It is reasonable to explain that large studies in AD have not demonstrated a meaningful increase in overall cancer risk, while also acknowledging that these data are indirect for SD and that uncertainty persists regarding rare outcomes such as CTCL [27-33]. Persistent, atypical, ulcerated, progressively infiltrated, or treatment-resistant lesions should be clinically reassessed rather than repeatedly treated empirically, particularly because inflammatory dermatoses and early CTCL may overlap in appearance.

Patient-Reported Outcomes and Quality of Life

Another important limitation of the current evidence base is its emphasis on clinician-assessed signs rather than outcomes that may be most meaningful to patients. Facial SD may be highly visible and recurrent, and its impact may therefore extend beyond erythema and scaling. Contemporary evidence suggests that SD can have an appreciable psychological and quality-of-life burden [3]. Yet, no direct tacrolimus study included in this review provided sufficient Dermatology Life Quality Index data to determine whether improvement in clinical severity translates into meaningful improvement in quality of life.

Patient preference was similarly poorly characterized. This matters because a maintenance treatment requiring intermittent long-term application may only be effective in routine practice if patients consider it acceptable, convenient, and worthwhile. Future trials should therefore assess validated quality-of-life measures, treatment satisfaction, adherence, treatment burden, cumulative corticosteroid exposure, and patient-reported symptom control alongside physician-assessed recurrence.

Strengths, Limitations, and Future Research

A strength of this review is the deliberate separation of direct SD efficacy evidence from indirect safety evidence. This reduces the risk of allowing the much larger AD literature to imply either SD-specific efficacy or SD-specific long-term safety. The review also distinguishes short-term induction from maintenance therapy and identifies overlap within the JOELLE cohort family, thereby avoiding treating analyses from the same underlying population as independent replication.

Several limitations remain. The review was not prospectively registered and did not have a publicly archived protocol. Only English-language reports were included, and the available audit trail did not allow confirmation of fully independent duplicate extraction and risk-of-bias assessment for every study. The outcome-level certainty assessment was added post hoc. Direct SD studies were generally small, varied substantially in regimen and outcome measurement, and rarely assessed quality of life. These limitations are consistent with concerns identified in earlier evidence syntheses, which have highlighted small samples, short follow-up, and inconsistent outcome reporting across SD trials [34,35].

The evidence is also insufficient to answer several clinically important questions. No eligible direct study provided separable data for immunocompromised patients or a clearly defined steroid-refractory population. Optimal duration of maintenance therapy remains uncertain, and it is not known whether benefit persists beyond the relatively limited follow-up periods studied in SD. Furthermore, although a twice-weekly regimen is supported by the most convincing direct evidence, the minimum effective maintenance frequency and the comparative cost-effectiveness of tacrolimus versus contemporary antifungal regimens remain uncertain.

Future randomized trials should therefore distinguish acute induction from proactive maintenance, use validated and standardized definitions of response and relapse, and include sufficiently long follow-up to evaluate durability. Comparisons with contemporary antifungal and low-potency corticosteroid strategies would be particularly valuable. Trials should also measure cumulative topical corticosteroid exposure, adherence, patient preference, and validated quality-of-life outcomes.

Direct SD studies large enough to resolve rare malignancy outcomes are unlikely to be feasible. Future long-term safety evidence may therefore depend on large registry or routinely collected healthcare datasets. Such studies should use appropriate active comparators, account for underlying disease severity and treatment indication, incorporate exposure latency, and perform analyses excluding malignancies diagnosed soon after treatment initiation to reduce the influence of protopathic bias. Until such evidence becomes available, long-term malignancy counseling in SD should remain appropriately cautious and should distinguish reassuring evidence regarding overall cancer risk from persisting uncertainty regarding rare diagnoses such as CTCL.

## Conclusions

Topical tacrolimus appears to be a useful off-label steroid-sparing option for selected patients with facial seborrhoeic dermatitis, with the strongest evidence supporting twice-weekly maintenance after disease control. Local burning or tingling is the most frequent adverse effect. Large AD studies are broadly reassuring for overall cancer risk, but they do not establish long-term malignancy safety in seborrhoeic dermatitis, and uncertainty remains around rare CTCL and lymphoma outcomes.
